# Copper requirements and copper toxicity as niche-defining factors in the growth of terrestrial ammonia-oxidizing archaea and bacteria

**DOI:** 10.1093/femsec/fiag026

**Published:** 2026-03-12

**Authors:** Barbora Oudova-Rivera, Andrew T Crombie, J Colin Murrell, Laura E Lehtovirta-Morley

**Affiliations:** School of Life Sciences, University of Warwick, Gibbet Hill Campus, Coventry CV4 7AL, United Kingdom; School of Biological Sciences, University of East Anglia, Norwich Research Park, Norwich NR4 7TJ, United Kingdom; School of Biological Sciences, University of East Anglia, Norwich Research Park, Norwich NR4 7TJ, United Kingdom; School of Environmental Sciences, University of East Anglia, Norwich Research Park, Norwich NR4 7TJ, United Kingdom; School of Life Sciences, University of Warwick, Gibbet Hill Campus, Coventry CV4 7AL, United Kingdom; School of Biological Sciences, University of East Anglia, Norwich Research Park, Norwich NR4 7TJ, United Kingdom

**Keywords:** nitrification, ammonia oxidising archaea, ammonia oxidizing bacteria, copper

## Abstract

Ammonia-oxidizing archaea (AOA) and bact eria (AOB) are critical for nitrogen cycling in the environment, but their copper requirements remain poorly understood. This study investigated copper requirements and toxicity thresholds for terrestrial AOA ‘*Ca*. Nitrosocosmicus franklandianus’ and *Nitrosotalea sinensis*, and the AOB *Nitrosomonas europaea* to understand the role of copper in their niche separation. Growth assays revealed significant differences between ammonia oxidisers in response to copper. *Nitrosotalea sinensis* exhibited the highest copper sensitivity, with full growth inhibition above 5 μM copper, while *N. europaea* demonstrated superior copper tolerance, withstanding up to 100 μM copper concentrations. Highest growth rates were observed at 50 nM copper for ‘*Ca*. N. franklandianus’ and at 10 nM for *N. sinensis*. Triethylenetetramine (TETA) provided protective effects against copper toxicity and enhanced the growth of both AOA strains by complexing inhibitory metals. Notably, hydroxylamine oxidation in AOA was inhibited by TETA, but it was not inhibited in AOB, indicating distinct differences in their metabolism. Cellular copper analysis confirmed that the amount of copper in cells differs between species. These findings demonstrate that copper availability and toxicity are important niche-differentiating factors for soil ammonia oxidisers. The differential copper sensitivities challenge current approaches using copper-chelating nitrification inhibitors.

## Teaser

Copper availability and toxicity differentially affect soil ammonia-oxidizing microbes, influencing their niche specialization and metabolic activity, with implications for understanding microbial diversity and nitrification inhibition strategies.

## Introduction

Copper is likely to play a significant role in ammonia oxidation and co-oxidation of other potential alternative substrates in ammonia-oxidizing archaea (AOA). Copper is involved in numerous physiological and metabolic cell functions of nitrifiers and methanotrophs (Semrau et al. 2010, Musiani et al. 2020). It is widely assumed, but not directly verified, that the archaeal ammonia monooxygenase (AMO) requires copper as a cofactor. This prediction is based on its similarity to other copper monooxygenases (CuMMOs) and its inhibition by a presumed copper chelator, allylthiourea (ATU) (Taylor et al. 2010, Lehtovirta-Morley et al. 2013, Gwak et al. 2020). Commercially available nitrification inhibitors, nitrapyrin, dicyandiamide (DCD), and ATU, inhibited ammonia oxidation in *Nitrosotalea devaniterrae* (Lehtovirta-Morley et al. 2013). However, ATU inhibited *Nitrosotalea devaniterrae* in liquid culture but was ineffective—or even slightly stimulatory—in soil microcosms, likely because soil reduced ATU bioavailability through adsorption or transformation, or because the archaeon’s acid-adapted physiology may make it less sensitive to the inhibitor under natural soil conditions (Lehtovirta-Morley et al. 2013). Non-linear dose responses and microbial community interactions may also contribute to the observed differences. (Lehtovirta-Morley et al. 2013). These inhibitors do not interact directly with AMO, but it is believed they act as copper chelators or, in the case of DCD, inhibit ammonia uptake (Powell and Prosser 1986, Lehtovirta-Morley et al. 2013). Copper appears to be fundamental to the electron-transfer machinery of AOA. Genomic analyses of model AOA such as *Nitrosopumilus maritimus* show that they lack the canonical cytochrome-c–based electron-transport systems typical of bacteria and instead encode an unusually large suite of copper-containing proteins, including blue-copper proteins and multicopper oxidases (Walker et al. 2010). The key enzyme initiating their chemolithotrophic pathway, ammonia monooxygenase (AMO), is itself a copper-dependent membrane monooxygenase, placing copper at the gateway of their respiratory chain (Musiani et al. 2020). Proteomic and structural studies further support a copper-centric electron-transfer system, suggesting that AOA rely on copper-based redox proteins instead of heme cytochromes (Reyes et al. 2020b, Hodgskiss et al. 2023). Collectively, these findings indicate that copper is not only important but is likely to be essential for the functioning of the AOA respiratory chain.

In ammonia-oxidizing bacteria (AOB), the respiratory chain is built around heme-based cytochromes, in contrast to the copper-centric system of AOA (Bergmann et al. 1994, Bergmann et al. 2005). Electrons generated during bacterial ammonia oxidation flow from hydroxylamine oxidoreductase (HAO) in the periplasm to the copper-dependent AMO, and then through a series of c-type and aa₃-type cytochromes, including cytochrome c₅₅₄, cytochrome c₅₅₂, and terminal oxidases such as cytochrome aa₃ oxidase (Dispirito et al. 1986, Bergmann et al. 1994, Bergmann et al. 2005). HAO is a particularly unusual multiheme enzyme: it contains multiple covalently-bound heme c groups (typically 7–8 hemes) arranged to support efficient intramolecular electron transfer (Arciero and Hoopers 1993, Igarashi et al. 1997, Bergmann et al. 2005). These hemes enable HAO to oxidise hydroxylamine to NO while simultaneously delivering electrons into the respiratory chain, where they contribute to the generation of a proton motive force and ATP synthesis (Arciero and Hoopers 1993, Igarashi et al. 1997, Bergmann et al. 2005, Caranto et al. 2017). AOA lack the bacterial hydroxylamine dehydrogenase (HAO) and canonical c-type cytochromes, suggesting they use alternative enzymes to oxidise ammonia intermediates. Genomic and proteomic studies indicate that multicopper oxidases (MCOs) and small blue-copper proteins may serve as functional analogues of HAO, facilitating hydroxylamine or nitroxyl oxidation and electron transfer to the quinone pool (Walker et al. 2010). Additionally, some AOA copper-containing nitrite reductases (NirK) may contribute to hydroxylamine oxidation or nitrogen turnover (Kobayashi et al. 2018), and F₄₂₀-dependent enzymes have been proposed as potential auxiliary electron carriers (Schatteman et al. 2022). These candidates highlight the possible unique, copper-centric electron transfer systems of AOA. It has therefore been speculated that AOA may have higher copper requirements than AOB, but this remains largely unexplored (Reyes et al. 2020a, Glass and Orphan 2012). Interestingly, the activity of AMO from *Nitrosomonas europaea* (AOB) was stimulated by the addition of copper, resulting in a 5- to 15-fold increase in ammonia-dependent O_2_ consumption, nitrite production, and hydrazine-dependent ethane oxidation (Ensign et al. 1993).

Ammonia oxidisers are often compared to methanotrophs because both groups oxidise structurally similar small substrates (methane or ammonia) using membrane-bound monooxygenases and share similar copper-dependent physiology and electron-transfer systems (Stein et al. 2012, Musiani et al. 2020). They are frequently used as model organisms because methanotrophs are much better understood than nitrifiers, particularly regarding the structure and function of particulate methane monooxygenase (pMMO), which has been used to infer aspects of the poorly understood AMO in nitrifiers (Stein et al. 2012, Musiani et al. 2020). Methanotrophs possess two types of methane monooxygenase: the copper-dependent pMMO, which dominates under high-copper conditions, and the iron-dependent soluble MMO (sMMO), expressed when copper is limiting (Murrell et al. 2000, Semrau et al. 2013). In methanotrophs that possess both types of methane monooxygenase, copper is essential for regulating the expression and activity of sMMO and pMMO, and in forming intracytoplasmic membrane stacks where pMMO is located. Crucially, the copper-to-biomass ratio is a more significant physiological indicator than the initial medium concentration, as rapid copper uptake by the cells can significantly deplete the available pool in the medium (Takeda and Tanaka 1980, Scott et al. 1981, Stanley et al. 1983, Murrell et al. 2000, Whiddon et al. 2019, Zhu et al. 2022). Methanotrophs also produce methanobactin, a copper acquisition molecule (Dispirito et al. 2016). A previous study demonstrated that methanobactin was secreted in the medium of the methanotroph *Methylococcus capsulatus* in copper-limiting conditions (≤10 µM Cu^2+^), presumably to provide cells with the essential copper, as well as in copper-excess conditions (≥60 µM Cu^2+^) to bind excess copper (Avdeeva and Gvozdev 2017). Because copper in soil is not uniformly available, and its bioavailability varies with pH, cation exchange capacity (CEC), organic–matter content and complexation chemistry, soil-dwelling micro-organisms are likely exposed to spatial and temporal fluctuations in copper stress—ranging from deficiency to toxicity (Dumestre et al. 1999, Kunito et al. 1999, Argüello et al. 2013). To survive, bacteria employ a range of adaptation and homeostasis mechanisms, such as transcriptional regulators, transporters, chaperones, and storage compartments (Argüello et al. 2013). These strategies allow soil bacteria to maintain cellular function and growth despite spatial and temporal variations in their environment. It is, therefore, likely that ammonia-oxidising archaea also possess some adaptation and homeostasis mechanisms for life in the soil, similar to their bacterial counterparts (Argüello et al. 2013) or copper acquisition molecules similar to methanobactin in methanotrophs (Dispirito et al. 2016), such as putative copper-storage protein Csps present in *Nitrososphaera viennensis* (Reyes et al. 2020b)

Copper is a naturally occurring element in soils, essential for all living organisms but potentially toxic at high concentrations. It mainly exists in soils as divalent copper within primary and secondary minerals and organic matter, with only a small fraction in soil solution as free Cu^2+^ or organic complexes (Mengel et al. 2001). Bioavailable copper concentrations in soil solution range from 1 × 10^−5^ to 6 × 10^−4^ mol/m^3^, and are influenced by pH and organic content (Sébastien Sauvé et al. 1997, Mengel et al. 2001). Copper deficiency is common in humus-rich soils where it binds strongly (McBride 1989).

Trace metal buffering is a common method used to regulate the bioavailability of trace metals, many of which are essential to cells but may become toxic in excess (Patton et al. 2004). Trace metal buffering works by adding chelating agents (like EDTA or citrate) that bind free metal ions, maintaining them at low but bioavailable concentrations, which prevents toxic spikes while ensuring sufficient availability for essential metalloenzymes in micro-organisms (Patton et al. 2004). Triethylenetetramine (TETA) is a potent metal chelator which selectively binds Cu^2+^. This compound has previously been utilised in studies related to the copper needs of AOA, including, for instance, marine AOA *Nitrosopumilus maritimus* (Amin et al. 2013) and terrestrial AOA *N. viennensis* (Reyes et al. 2020a).

The specific growth rate of *Nitrosopumilus maritimus*, a marine AOA, is affected by copper concentration both when its concentration is limiting and toxic (Amin et al. 2013). Transcriptional analysis revealed the downregulation of genes encoding for the 3-Hydroxypropionate/4-hydroxybutyrate (HP/HB) cycle and upregulation in the biosynthetic pathway of cobalamin in both limiting and toxic conditions, compared to control (Amin et al. 2013, Qin et al. 2017).

The growth of the terrestrial AOA, *N. viennensis*, was limited when the total copper was below 1 nM, estimated to correspond to 6 fM free Cu^2+^ (Reyes et al. 2020a), a form that is utilized by AOA (Amin et al. 2013). In response to copper limitation compared to standard Cu-replete medium, the transcriptome of *N. viennensis* showed a differential expression of half of all detected genes. Among the upregulated genes were those encoding proteins involved in copper binding, transport, and uptake. Downregulated genes were linked to ammonia oxidation, carbon uptake, and biosynthesis (Reyes et al. 2020b).

A study conducted on microbial cultures from wastewater treatment plants also suggested copper limitation of selected nitrifiers, including AOA. In this case, copper was complexed with organic matter, and the addition of Cu^2+^ alleviated the inhibition of *N. viennensis* (Gwak et al. 2020).

Higher dependency of AOA rather than AOB on copper, as proposed before (Reyes et al. 2020a, Glass and Orphan 2012), would explain the transcriptional response upon copper limitation. The differential requirements of AOA and AOB for copper and their physiological response to copper-induced stress within environmentally relevant copper concentration conditions likely shape the niche distribution of ammonia-oxidizing microbes (Reyes et al. 2020a, Glass and Orphan 2012, Amin et al. 2013, Gwak et al. 2020, Shafiee et al. 2021). AOA seem to have a higher affinity for copper than AOB (Gorman-Lewis et al. 2019), and their growth is also more limited in copper-deplete conditions (Gwak et al. 2020). However, there are only a handful of studies on the copper requirements of ammonia oxidizers, and more evidence is needed to understand the differences between nitrifying guilds and how they respond to Cu^2+^.

The aim of this study was to determine whether terrestrial AOA and AOB have different requirements for copper. Specifically, this study set out to answer: 1. What are the copper requirements and toxicity thresholds of AOA ‘*Ca*. Nitrosocosmicus franklandianus’ and *Nitrosotalea sinensis*, and AOB *N. europaea*? 2. How much copper do AOA and AOB cells contain? and 3. How tightly is copper bound in the ammonia and hydroxylamine oxidation machinery in AOA and AOB?

## Methods

### Preparation of metal-limited media

*‘Ca*. N. franklandianus’ was maintained in fresh water medium (FWM) (Lehtovirta-Morley et al. 2016) static incubator at 37°C. *Nitrosomonas europaea* was maintained in the FWM at 30°C static. The acidophilic AOA *N. sinensis* was maintained at 37°C in FWM medium buffered to pH 5.3 with MES and 4 mM NaHCO_3_ as previously described (Lehtovirta-Morley et al. 2011). Prior to use in experiments, cultures were transferred into copper-free medium and sub-cultured twice to reduce the presumed internal storage of copper. To limit copper availability in ammonia-oxidizing cultures, several steps were taken to prevent contamination of the media by metals from glassware and media components. All media components were prepared and stored exclusively in polystyrene vacuum bottles (Nalgene® vacuum filtration system, vol. 1000 ml, pore size 0.2 μm). Cultures were maintained in Corning® square polycarbonate storage bottles (150 ml) without added copper, in order to reduce their intracellular copper stocks. All culturing bottles were soaked for 3 days in 10% nitric acid at 37°C, then rinsed with ultrapure water and sterilized in a clean autoclavable bag in the microwave for 5 min at 1000 W. All media components (except metals and chelators) were mixed with 0.5 g l^−1^ or 5 g l^−1^ (depending on treatment) Chelex® 100 sodium form (Sigma-Aldrich) and stirred for 1 h to remove contaminant trace metals, then decanted and filter-sterilized. The manufacturer’s general recommendation is to use 50 g l^−1^ resin, but this amount appeared excessive, as it caused a drastic drop in pH (<3), inhibiting the growth of all tested model ammonia oxidizers. In addition, although the Chelex resin has a high affinity for binding copper, it can also bind other cations, including calcium, magnesium and sodium, which are important media components in this experimental system. Therefore, to remove Cu^2+^ while maintaining other cations, the concentration of Chelex resin needed to be reduced. Using 5 g l^−1^ of Chelex resin removed a similar amount of copper as 50 g l^−1^ (Table S1) and did not inhibit the growth of model micro-organisms.

The copper chelator TETA (triethylenetetramine) was added to the culture medium in order to buffer free Cu²⁺ levels, because TETA has a very high binding affinity for Cu(II) over other divalent metals. Potentiometric and spectrophotometric studies showed that TETA forms very stable 1 : 1 complexes with Cu²⁺ (with Cu values that greatly exceed those for Zn²⁺) (Nurchi et al. 2013), and other biological studies (Reyes et al. 2020a, Amin et al. 2013) have used TETA as a highly specific divalent copper chelator in microbial assays. By acting as a high-affinity ligand, TETA allows for the maintenance of stable, bioavailable Cu^2+^ concentrations at the picomolar or femtomolar level, even in the presence of higher total copper. This buffering capacity effectively mimics the conditions found in natural soil environments, where Cu^2+^—the most common species available for microbial uptake—is largely complexed by organic matter, yet remains accessible at trace levels. TETA is also clinically used in Wilson’s disease to chelate excess copper and promote its urinary excretion, thereby preventing toxic copper accumulation in tissues (Herzog et al. 2019). The copper chelator TETA (Triethylenetetramine hydrate, 98%, Sigma–Aldrich) was used in the culture media at 1 mM or 10 mM concentrations according to treatment. Copper(II) chloride, 99%, extra pure, anhydrous, was purchased from Acros Organics.

Growth assays were performed in 150 ml bottles (Corning® square polycarbonate storage bottles) in triplicate. The accumulation of nitrite was measured daily until the cultures reached the stationary phase. Activity assays were performed in 30 ml Universal containers (Greiner Bio) in triplicate. The biomass was harvested at mid-exponential phase with a vacuum filtration system using 0.22 PES membrane filters and resuspended in salt solution and buffer (MES for *N. sinensis* or HEPES for *N. europaea* and ‘*Ca*. N. franklandianus’). The cell suspension was then left in the incubator for 1 h to allow endogenous respiration to cease. CuCl_2_, TETA, NH_4_Cl, and NH_2_OH were added according to the treatment. Nitrite accumulation in growth and activity assays was determined using Griess reagent in a 96-well plate format as described before (Oudova-Rivera et al. 2023).

### Trace metal determination

Trace metal concentrations in liquid and biomass samples were analyzed in the Mass spectrometry platform at the University of East Anglia by ICP-MS-QQQ in single and triple quad mode. The limit of quantification for copper was 0.357 µg/kg. The cultures were grown in standard medium with 10 nM CuCl_2_ in sterile, acid-washed glass bottles in triplicate. The volume of *N. europaea* was 500 ml per sample, ‘*Ca*. N. franklandianus’ 1 l per sample and *N. sinensis* 2 l per sample. Biomass samples were harvested by filtration during mid-exponential phase, washed three times and resuspended in 100 μl analytical grade water (Fisher Chemical^TM^). Chemical speciation was modelled using Visual MINTEQ 3.1 with inbuilt stability constant database. TETA stability constants were added manually (Anderegg et al. 2005). Statistical analysis was performed using IBM SPSS Statistics for Macintosh, Version 27.0. Significant differences in maximum growth rates between copper treatments and control groups were determined using a one-way analysis of variance (ANOVA) followed by Tukey’s post-hoc test for multiple comparisons (*P* < 0.05).

## Results

### Optimizing the experimental set-up to limit available copper

The standard concentration of copper added to growth medium for *N. sinensis*, ‘*Ca*. N. franklandianus’ and *N. europaea* is 10 nM CuCl_2_ (Lehtovirta-Morley et al. 2011, 2016). However, the actual copper concentration is usually higher because copper is a contaminant present in small amounts in other medium components and in glassware. Washing or soaking glassware with acid was ineffective at removing nanomolar traces of copper, possibly because autoclaving could be a source of metal contamination (Table S1). Medium prepared in acid-washed glassware contained as much as 410 µg l^−1^ iron, 13 µg l^−1^ copper (∼203 nM), and 34 µg l^−1^ zinc without the addition of these metals (Table S1). Since it was not possible to fully prevent copper contamination from glass bottles, all experiments were conducted in polystyrene or polycarbonate bottles and media were filter-sterilized as described in the method section.

#### Determination of growth-limiting and toxic copper thresholds in selected ammonia oxidizers

To determine how copper availability affects the physiology of AOA and AOB, we conducted controlled growth experiments with *N. sinensis*, ‘*Ca*. N. franklandianus’ and *N. europaea* across a wide range of copper concentrations and media conditions. Copper is considered an essential cofactor for AMO, and its bioavailability is strongly influenced by pH, complexation, and background trace-metal chemistry. ‘*Ca*. N. franklandianus’ is an ammonia-oxidizing archaeon isolated from fertile arable soil that grows optimally at neutral to slightly alkaline pH, making it a representative model of non-acidophilic soil AOA (Lehtovirta-Morley et al. 2016). *Nitrosotalea sinensis* is a particularly informative model organism for these experiments because it is an acidophile (Lehtovirta-Morley et al. 2011), and low pH environments can markedly alter the speciation and bioavailability of copper, potentially shifting growth thresholds and toxicity limits (Hasman et al. 2009). To disentangle these effects, we compared growth in standard medium, copper-buffered medium containing the Cu-specific chelator TETA, and copper-depleted medium treated with Chelex resin. The following section describes how these contrasting copper regimes influenced growth rates, lag phases, and nitrite production in all species.

#### Growth-limiting and toxic copper thresholds of *N. sinensis*

**Figure 1:**
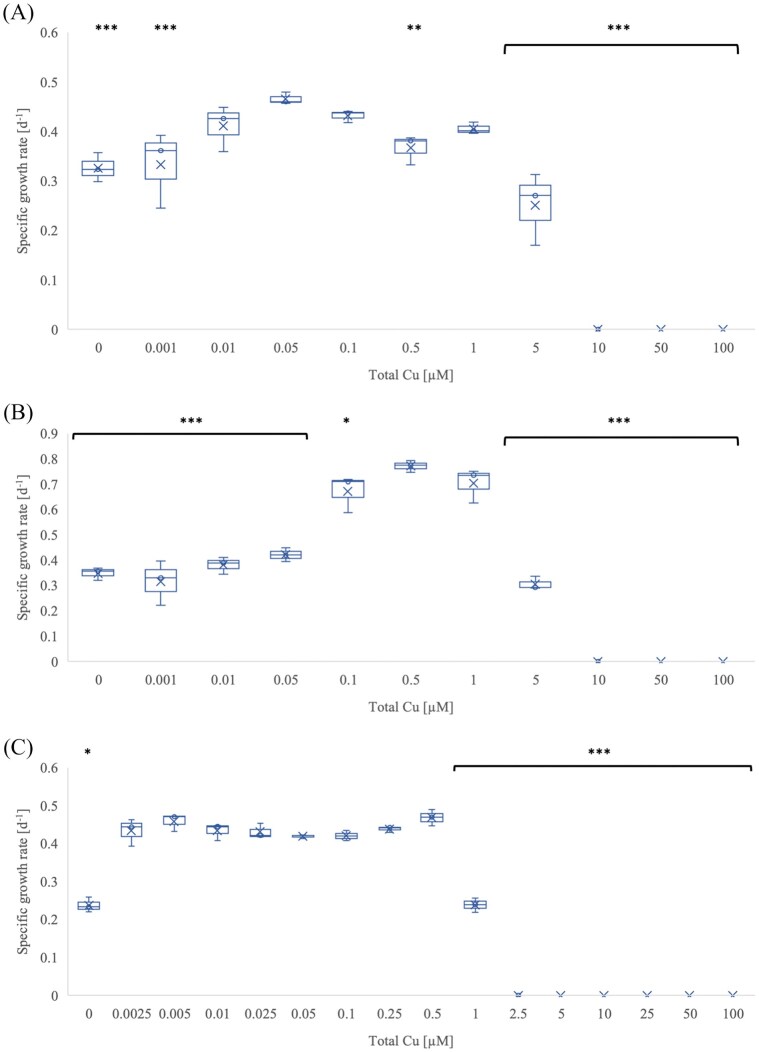
The effect of copper on the specific growth rate of *N. sinensis*. (A) Standard medium; (B) medium with 1 µM TETA; (C) medium treated with Chelex resin. Total copper represents the concentration of copper added to the medium. Error bars represent standard error (*n* = 3). *P*-values compare the treatment with the highest growth rate with other treatments; * *P* ≤ 0.05; ** *P* ≤ 0.01; *** *P* ≤ 0.001.

The optimal concentration of copper for the growth of *N. sinensis* in standard medium (FWM without TETA and not treated with Chelex resin) (Fig. 1A) was 50 nM, resulting in a specific growth rate of 0.47 ± 0.007 d^−1^. Copper concentrations ≤ 1 nM significantly reduced the specific growth rate to 0.32 ± 0.01 d^−1^ (no added copper) and 0.33 ± 0.04 d^−1^ (1 nM added copper). Five hundred nanometres copper was growth limiting, and concentrations higher than 5 µM were toxic.

The optimal copper concentration for the growth of *N. sinensis* with 1 µM TETA (Fig. 1B) was 500 nM. With this copper concentration in the medium *N. sinensis* had a specific growth rate of around 0.77 ± 0.02 d^−1^, which was considerably higher than in the standard medium. Copper concentrations of 100 nM or less were limiting, while 5 µM copper was toxic (Fig. 1B).

When grown in medium treated with Chelex resin (Fig. 1C) without any added copper, the specific growth rate was reduced by ∼50%, from 0.46 ± 0.02 d^−1^ to 0.24 ± 0.01 d^−1^ compared to the treatment with 5 nM copper. The lag phase (Supplementary fig. 1) doubled under copper limitation, from 6.3 (± 0.3) days to 12.6 (± 2.6) days, and the concentration of accumulated nitrite (after 49 days) was only 86 ± 32 µM compared to 434 ± 7 µM in the presence of 10 nM added copper. There was no significant difference in any of these parameters between treatments with an added copper concentration between 2.5 and 500 nM. 1 µM copper concentration led to a specific growth rate of 0.23 ± 2.92 d^−1^, with an extended lag phase of 19 ± 0.3 days (Supplementary Fig. 1) and low nitrite yield (36 ± 26 µM). The standard nitrite yield of a healthy and uninhibited culture was around 400–500 µM before reaching the stationary phase.

#### Growth-limiting and toxic copper thresholds for ‘*Ca*. N. franklandianus’

**Figure 2:**
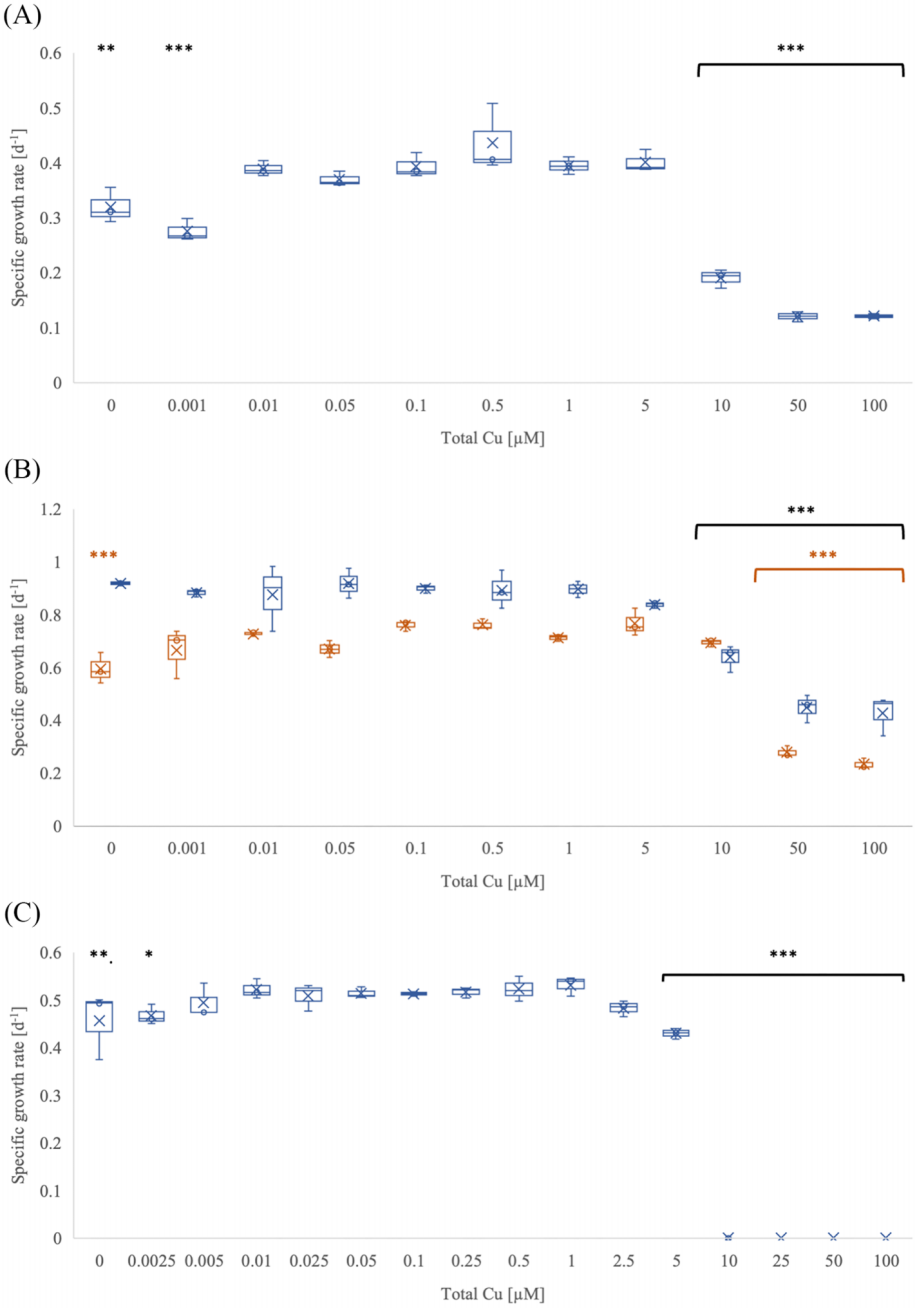
The effect of copper on the specific growth rate of '*Ca*. N. franklandianus'. (A) Standard medium; (B) Medium with 1 µM TETA (blue) and 10 µM TETA (orange); (C) Medium treated with Chelex resin. Total copper represents the concentration of copper added to the medium. Error bars represent standard error (*n* = 3). *P*-values compare the treatment with the highest growth rate with other treatments; * *P* ≤ 0.05; ** *P* ≤ 0.01; *** *P* ≤ 0.001.

*Ca*. N. franklandianus' was first grown in standard medium (not treated with Chelex, without TETA), supplied with a range of copper concentrations (1 nM–100 µM) (Fig. 2A). The optimal copper concentration was 10 nM, with a specific growth rate of 0.38 ± 0.01 d^−1^. There was no significant difference in the growth rate in treatments with copper concentration between 10 nM and 5 µM. Treatments with no added copper and 1 nM copper resulted in a significantly lower specific growth rate of 0.32 ± 0.02 and 0.28 ± 0.01 d^−1^. More than 10 µM copper was toxic, lowering the specific growth rate significantly. The final nitrite yield was reduced by 84% at 10 µM copper concentration and by > 94% at 50 µM and 100 µM copper concentration over ten days.

In experiments with ‘*Ca*. N. franklandianus’ grown in medium with TETA (Fig. 2B), 1 µM and 10 µM TETA concentrations were used to limit the available copper concentration because the growth rate was not affected with 1 µM TETA. 10 µM TETA was more effective in slowing the growth of ‘*Ca*. N. franklandianus’ without added copper, but only by ∼20%, and major growth limitation was not achieved. The specific growth rate was significantly lower in the treatment without additional copper, 0.59 ± 0.03 d^−1^, compared to 0.72 ± 0.01 d^−1^ in the treatment with 10 nM copper. The total nitrite yield was reduced to 2189 ± 59 µM and 2087 ± 141 µM in treatments with 0 and 1 nM added copper over 19 days, compared to 3165 ± 68 when supplied with 10 nM total copper and 10 µM TETA. In the medium with 1 µM TETA, ≥ 10 µM copper was toxic, and with 10 µM TETA, copper ≥ 50 µM was toxic, resulting in a reduction in specific growth rate and reduced total nitrite yield (≥ 95%). Considering the TETA: Cu binding ratio of 1 : 1, the effect of TETA was limited in conditions where the copper concentration was higher than the concentration of TETA.

In medium treated with 5 g l^−1^ chelex resin (Fig. 2C), the specific growth rate was slightly reduced in the absence of added copper (∼12%) and with 2.5 nM copper (∼10%) compared to 10 nM copper (0.52 ± 0.01 d^−1^). The lag phase was not affected in these treatments (∼6 days), nor was the final nitrite yield after 49 days. 5 µM copper concentration led to a lower specific growth rate (0.43 ± 0.01 d^−1^) and longer lag phase (9.6 ± 0.6 days), and ≥ 10 µM copper caused complete inhibition of nitrite production.

#### Growth-limiting and toxic copper thresholds of *N. europaea*

**Figure 3:**
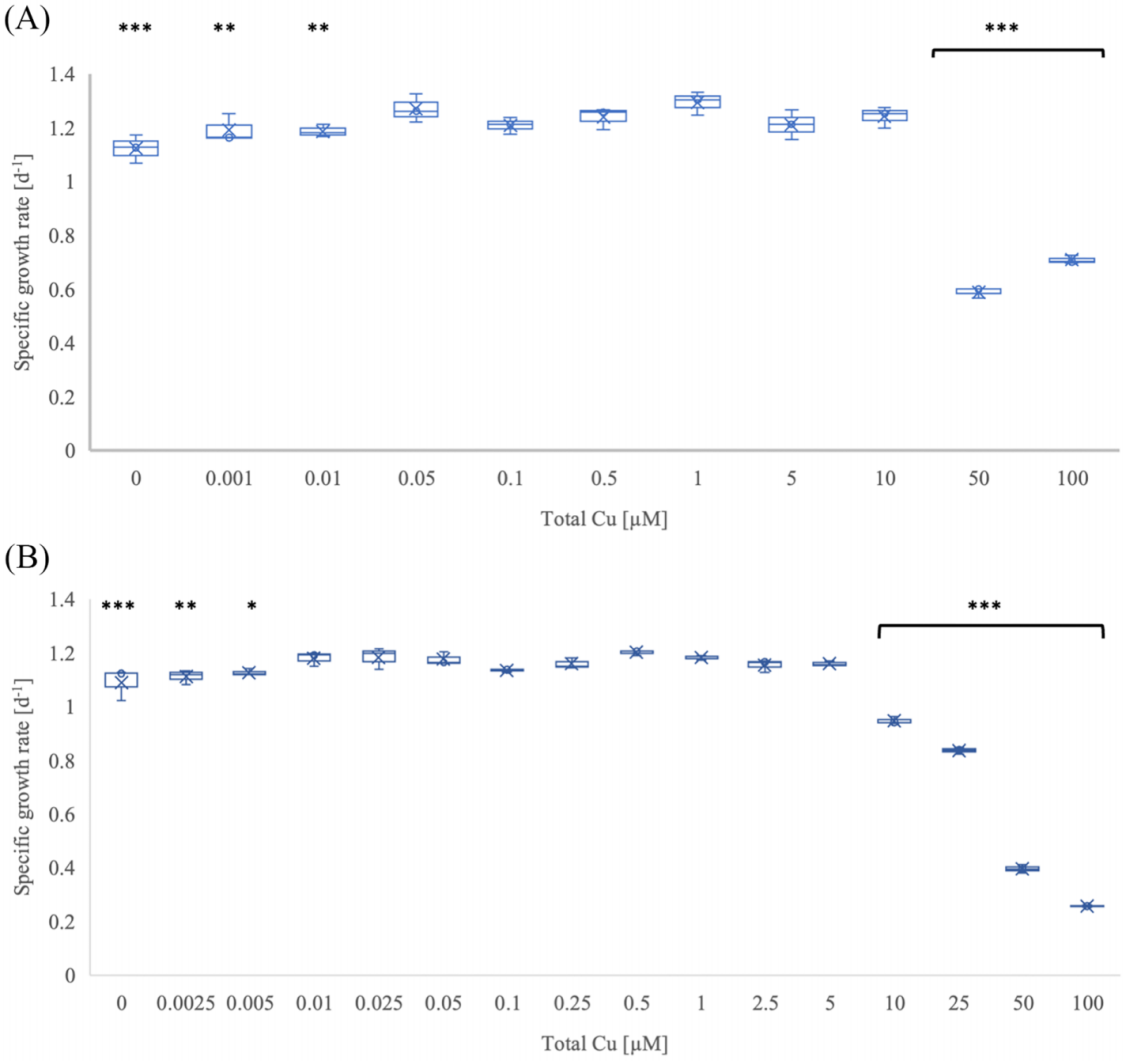
The effect of copper on the specific growth rate of *N. europaea*. (A) Medium with 1 µM TETA; (B) Medium treated with Chelex resin. Total copper represents the concentration of copper added to the medium. Error bars represent standard error (*n* = 3). *P*-values compare the treatment with the highest growth rate with other treatments; * *P* ≤ 0.05; ** *P* ≤ 0.01; *** *P* ≤ 0.001.

Since previous experiments showed that the standard medium did not adequately control copper availability in the two archaeal strains, we cultivated *N. europaea* directly in Chelex-treated and TETA-supplemented medium to ensure consistent and tightly regulated copper concentrations across all experiments*. Nitrosomonas europaea* (AOB) was first grown in medium containing 1 µM TETA (Fig. 3A). The optimal copper concentration was 50 nM, with a specific growth rate of 1.27 ± 0.03 d^−1^. There was no significant difference in the specific growth rate with the copper concentration between 50 nM and 10 µM, which is a broader range of copper concentrations at which *N. europaea* grows optimally, compared to the studied AOA strains, that have a narrower optimal range. Lower concentrations of copper led to a slightly reduced growth rate (<8%), but there was no significant difference in the total nitrite yield. Copper concentrations ≥ 50 µM were toxic.

The optimal concentration of copper in the medium treated with 5 g l^−1^ chelex resin was 10 nM (Fig. 3B). The addition of copper in concentrations between 0 and 5 nM led to a slightly reduced specific growth rate. However, there was no significant difference in the duration of the lag phase and final nitrite yield. Copper concentrations between 10–50 µM were toxic and reduced the specific growth rate, but there was no significant difference in the total nitrite yield. The average lag phase was < 1 day in treatments with ≤ 10 µM copper, 1.3 ± 0.3 days in 25 µM copper, 5 ± 0.6 days in 50 µM copper, and 20.6 ± 1.6 in 100 µM copper (Supplementary fig. 1).

### The effect of copper on the oxidation of ammonia and hydroxylamine by ammonia-oxidizing micro-organisms

**Figure 4:**
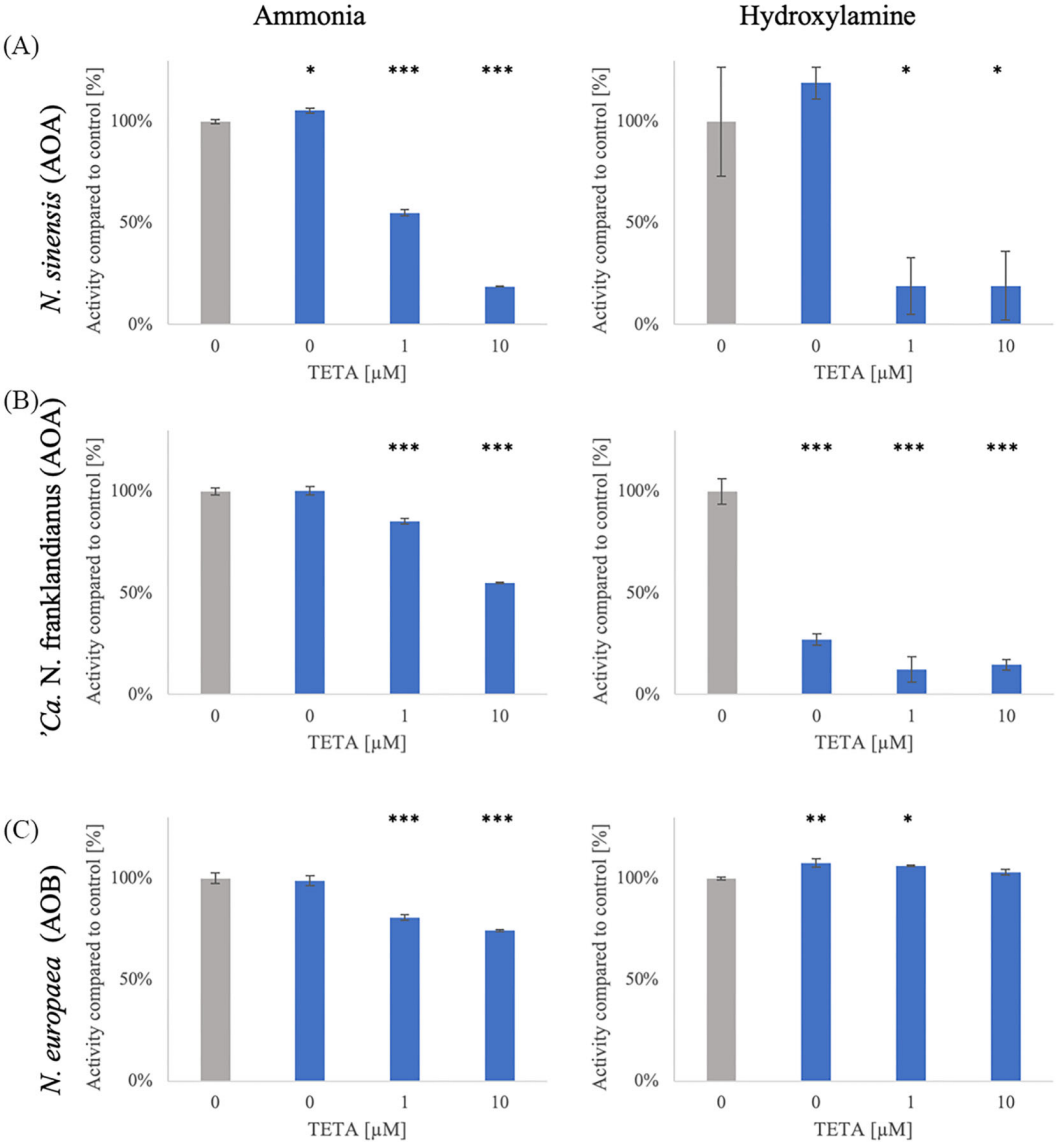
The effect of copper chelator TETA on ammonia and hydroxylamine oxidation by ammonia oxidizers. TETA was applied as 0, 1 µM or 10 µM. 10 nM copper was added to the control (grey), and no copper was added in TETA treatments (blue). Hydroxylamine and NH_4_Cl were applied at 200 µM concentration. Error bars represent standard error (*n* = 3). *P*-value compares treatments to the control; * *P* ≤ 0.05; ** *P* ≤ 0.01; *** *P* ≤ 0.001.

The importance of copper varies among ammonia oxidizers. While the ammonia monooxygenase in both AOA and AOB most likely relies on copper as a cofactor, the downstream pathway for ammonia oxidation is different (Lehtovirta-Morley 2018). To test how tightly copper is bound to the components of the ammonia and hydroxylamine oxidation machinery, an activity assay was performed. Pure cultures of three model strains of AOA and AOB were harvested, diluted to 100 ng protein ml^−1^ and supplied with either ammonia or hydroxylamine and different concentrations of the copper chelator TETA. Controls were supplied with an additional 10 nM copper. Nitrite accumulation was measured every 15 min for 1 h. The results (Fig. 4) demonstrate a difference between archaeal and bacterial ammonia oxidizers. The activity (measured as nitrite accumulation) of *N. sinensis* supplied with ammonia (Fig. 4A) decreased to 55.1 ± 1.2% and 18.8 ± 0.2% of the control with the addition of 1 µM and 10 µM TETA, respectively. With hydroxylamine as substrate, the activity decreased to 19.1 ± 14.1 and 19.1 ± 17% of the control in the presence of 1 µM and 10 µM TETA, respectively.

Ammonia oxidation activity of ‘*Ca*. N. franklandianus’ (Fig. 4B) was reduced by 14.5 ± 1.4% and 45 ± 0.3% with the addition of 1 and 10 µM TETA, respectively, compared to the control. The hydroxylamine oxidation was reduced in the absence of additional copper (without TETA) by 73 ± 2.7%. With the addition of 1 and 10 µM TETA, the hydroxylamine oxidation was reduced by 87.7 ±6.3% and 85.4 ± 2.7%, respectively.

The activity of *N. europaea* (AOB) (Fig. 4C) supplied with ammonia (same concentration) was reduced by the addition of TETA. 1 µM TETA reduced the activity to 80.6 ± 1.5%, and 10 µM TETA to 74.2 ± 0.6%. The absence of additional copper and the addition of 1 µM TETA caused a small, but significant increase in the ammonia and hydroxylamine oxidation activity (7.6 ± 2.1% and 6.1 ± 0.2%, respectively, Fig. 4C).

Interestingly, hydroxylamine oxidation by both AOA strains was more strongly inhibited at a lower concentration of TETA compared to ammonia oxidation. The opposite trend was observed with AOB, where ammonia oxidation was inhibited with TETA, but hydroxylamine oxidation was not. This consistent with the current understanding that the hydroxylamine oxidation by AOB it is not dependent on copper, but ammonia oxidation is. Intriguingly, the inhibition of archaeal hydroxylamine oxidation by TETA suggests that the unidentified enzyme(s) involved in this pathway may be copper-dependent.

### Copper content of cells of ammonia oxidizers

To understand the differences in the response of ammonia-oxidizing micro-organisms to copper-limited conditions, we quantified the copper content in the biomass of three model nitrifiers (‘*Ca*. N. franklandianus’, *N. sinensis*, and *N. europaea*) using ICP-MS. Copper is an essential cofactor for ammonia monooxygenase and other key enzymes, and we expected that differences in copper demand or accumulation might reflect adaptations to environmental niches and growth physiology. By analyzing cultures grown in medium without TETA and not treated with Chelex, we aimed to compare both cellular copper quotas and copper concentration per unit protein, anticipating that acidophilic and neutral-pH AOA and AOB might differ in their total copper content due to variations in cell size, metabolic rate, and enzyme copper requirements.

The metal content of the biomass of the three model ammonia oxidizers was quantified using ICP-MS. All three cultures were grown in medium without TETA or Chelex treatment. The copper content in the biomass of *N. europaea* was 2.16 (±0.12) mg g^−1^ of protein, which was the highest of the three analyzed nitrifiers. The lowest concentration was found in *N. sinensis*, 0.21 (±0.01) mg g^−1^ of protein. ‘*Ca*. N. franklandianus’ had 1.10 (±0.28) mg g^−1^ of protein. Considering that the size of cell of ‘*Ca*. N. franklandianus’ (555 fg of protein per cell) is significantly larger than that of *N. sinensis* (80 fg of protein per cell) and *N. europaea* (120 fg of protein per cell), the amount of copper per cell was highest in ‘*Ca*. N. franklandianus’.

### Copper speciation modelling

Studies previously performed on the copper requirements of ammonia-oxidizing archaea reported the total added copper concentrations, as well as the concentration of free Cu^2+^ (Reyes et al. 2020a, Amin et al. 2013). The concentrations of free Cu^2+^ were estimated using chemical speciation modelling software (MINEQL and PhreeqC, respectively). Copper speciation in this study was performed using the visual MINTEQ software package (Gustafsson 2004). The concentration of free Cu^2+^ in the medium with 10 nM added copper concentration was 42.4 fM in the medium for *N. europaea* and ‘*Ca*. N. franklandianus’, and 110 fM in the medium for *N. sinensis* (Supplementary material, Table 2).

However, chemical speciation software estimates the proportion of each chemical species using a set of experimentally obtained thermodynamic constants available in the literature. The proportion of all species is affected by pH, temperature, concentrations of all trace elements and affinities of all acids for metals in the medium. Also, nitrite accumulation in the media of growing ammonia oxidizers leads to gradually decreasing pH. Considering that each constant has a certain error and that additional errors are introduced in medium preparation, the actual concentration of a cation of interest might drastically differ from the one predicted by the model. The concentration of copper could be reliably controlled, as well as predicted by speciation models, if the concentration of EDTA in the medium was more than 10 000 × higher than the concentration of all trace elements in the medium, including iron. The EDTA would act as a buffer, leading to an equilibrated medium. The media used for the growth of ammonia oxidizers are not optimized for trace metal work, and therefore, the concentration of free copper cannot be reliably controlled (Sunda et al. 2005).

## Discussion

The ideal copper concentrations for the growth of *N. sinensis* and *N. europaea* in the chelated (TETA) medium were 500 nM and 50 nM, respectively (Figs. 1B and 3A). Additional copper did not significantly affect the specific growth rate of ‘*Ca*. N. franklandianus’ (Fig. 2B). In comparison, the optimal copper concentrations for *N. viennensis* and *N. maritimus*, were 5 nM and 50 nM, respectively (Reyes et al. 2020a, Amin et al. 2013). Interestingly, both AOA strains (*N. sinensis* and ‘*Ca*. N. franklandianus’) had a higher specific growth rate in the presence of 1 µM TETA compared to experiments without any TETA. It is possible that this was because TETA formed complexes with other metals, such as nickel, zinc, or cobalt, which may have been inhibitory for AOA (Lachowicz et al. 2019). While these metals are essential at trace levels, they can become inhibitory when they outcompete copper for binding sites in proteins or interfere with the assembly of metal centres in enzymes unique to AOA. For instance, the AOA respiratory chain relies on a high number of plastocyanins and other blue-copper proteins. An excess of other divalent cations could result in the mismetallation of these proteins, rendering them non-functional (Foster et al. 2022). The ability of TETA to complex a range of divalent cations (Lachowicz et al. 2019), although much less specifically compared to copper, may effectively lower the background toxicity of the medium, creating a more favourable conditions for the sensitive AOA strains. The higher growth rate in the TETA-supplemented medium can also be attributed to the way TETA buffers free Cu²⁺ and stabilises copper speciation. In medium without TETA or Chelex treatment, added copper partitions among free Cu²⁺, hydrolyzed species, and complexes with inorganic ligands (e.g. carbonate, phosphate) or organic matter, leading to strong pH-dependent shifts in bioavailable copper and a narrow margin between limitation and toxicity (Sunda et al. 2000, Price and Morel 2005, Morel et al. 2025). The toxic effect of copper was more pronounced in media treated with Chelex resin compared to the standard media (Figs. 1 A, C and 2 A, C). It is possible that the Chelex treatment, while effective at removing copper, also removes other medium components or trace organics that provide natural buffering capacity. If these components act as copper chelator, removing them would result in a higher proportion of free, bioavailable Cu^2+^ in the Chelex-treated media than in standard media at the same total copper concentration. Furthermore, extensive depletion of other essential divalent cations by Chelex may exacerbate cellular stress, reducing the physiological capacity of micro-organisms to manage copper toxicity (Sunda et al. 2005). At low total copper, micro-organisms can therefore experience functional copper limitation, while moderate increases can rapidly elevate free Cu²⁺ to inhibitory levels.

In contrast, adding TETA creates a metal-buffered system in which most copper is held in highly stable Cu–TETA complexes, maintaining a small, steady pool of free Cu²⁺ that supports copper-dependent metabolism without reaching toxic concentrations (Nurchi et al. 2013). This allows the organisms to access sufficient copper at higher total concentrations, resulting in a considerably higher growth rate (0.77 ± 0.02 d⁻¹) compared to medium without TETA. While dynamic factors such as medium acidification and biomass-associated copper uptake may influence free ion availability during the later stages of growth, the pronounced differences in the maximum specific growth rate​ between AOA and AOB in identical buffering conditions suggest distinct physiological responses to copper availability. AOA likely possess distinct metal-acquisition strategies or higher requirements for copper-buffered environments to maintain their extensive suite of copper-containing respiratory proteins. TETA also had a significant protective effect against copper toxicity, as both AOA and AOB could tolerate higher concentrations of copper with TETA in the medium. Thus, routine addition of TETA into the growth medium for ammonia oxidizers should be considered.

Growth of *N. sinensis* was considerably lower when copper concentration was below 10 nM and below 500 nM in the presence of 1 µM TETA (Fig. 1A, B). This is consistent with genomic predictions for other ammonia-oxidizing archaea, which suggest a heavy reliance on copper due to an abundance of copper-containing metabolic enzymes (Walker et al. 2010). Moreover, copper chelation by TETA likely intensifies this limitation by reducing free Cu²⁺, mirroring previous observations that AOA growth is strongly constrained under copper-limited or chelator-amended conditions (Amin et al. 2013, Reyes et al. 2020a). The toxicity thresholds were the lowest among all tested ammonia oxidizers. The relatively high copper requirements of *N. sinensis* could potentially be explained by the increasing availability of free Cu^2+^ at pH < 5. Empirical relationships suggest that free Cu²⁺ activity may increase by approximately two orders of magnitude for each unit decrease in pH in this range (Sébastien Sauvé et al. 1997). However, the range of copper concentrations that support growth (neither limiting nor toxic) appears somewhat narrow compared to other terrestrial micro-organisms, but similar to the range observed in *N. maritimus* (marine AOA) (Amin et al. 2013). In contrast to other ammonia oxidizers tested in this study, complete growth inhibition was observed at the highest copper concentrations for *N. sinensis* (Fig. 1). In addition, the cellular copper content in *N. sinensis* was very low, suggesting that it does not store much copper and may lack sufficient homeostasis mechanisms to manage fluctuating copper levels. Interestingly, a liquid culture of ‘*Nitrosotalea devaniterrae*, from the same genus as *N. sinensis*, was more sensitive to the copper-chelating nitrification inhibitor ATU than other tested AOA (Lehtovirta-Morley et al. 2013, Gwak et al. 2020).

Growth of ‘*Ca*. N. franklandianus' was only slightly affected in copper-deplete medium, possibly because its cellular copper content was the highest of the three tested ammonia oxidizers. A larger reduction in growth or final nitrite yield, as seen in *N. sinensis* or previously in *N. viennensis* (Reyes et al. 2020a) was not observed. Note that experiments with *N. viennensis* were conducted in a trace metal-free clean room with a HEPA filtration system (Reyes et al. 2020a). Such a setting was not available for the experiments in this study, and it remains unclear whether ‘*Ca*. N. franklandianus’ would become more copper-limited under those conditions.

The specific growth rate of the bacterial ammonia oxidizer, *N. europaea*, was only slightly reduced at low copper concentrations, and this strain could tolerate 50–10 000 nM copper in the presence of TETA (1 µM) (Fig. 3A).

The response to high copper concentrations varied among the tested strains. For *N. europaea*, the toxic inhibitory threshold was similar to that of ‘*Ca*. N. franklandianus’ (10 µM in the presence of 1 µM TETA), and *N. sinensis* was the most sensitive (threshold 1 µM). Furthermore, *N. sinensis* was fully inhibited above its threshold, whereas ‘*Ca*. N. franklandianus' maintained some growth (except in the experiment with Chelex-treated medium) above its inhibitory threshold and sustained low but steady activity, however, the final nitrite yield was dramatically reduced. It was remarkable that *N. europaea* grew even at the highest concentration of 100 µM copper. The lag phase was significantly prolonged, yet the final nitrite yield was consistent with other treatments. This suggests that *N. europaea* possesses mechanisms to cope with copper toxicity, which could have been induced during the extended lag phase. Previous reports indicate that copper toxicity in *N. europaea* increases with rising ammonia concentrations, presumably due to the formation of toxic copper-amine complexes (Sato et al. 1988). The pronounced sensitivity of AOA to elevated copper likely stems from the susceptibility of their unique metabolic machinery. In many microbes, copper toxicity is driven by the production of reactive oxygen species (ROS) via Fenton-like reactions, which cause oxidative damage to lipids, DNA, and proteins (Dupont et al. 2011). Furthermore, copper can displace iron from iron-sulfur (Fe-S) clusters in essential dehydratases, leading to the inactivation of key metabolic pathways (Macomber and Imlay 2009). In ammonia oxidizers, where many predicted enzymes for ammonia oxidation and carbon fixation rely on metal centres, such displacement could be particularly disruptive.

A notable difference between AOA and AOB was also evident in their dependence on copper for the hydroxylamine oxidation pathway (Fig. 4). No significant reduction in hydroxylamine oxidation activity was expected in *N. europaea* (AOB) across any treatment, since its downstream ammonia oxidation pathway is iron-dependent (Stahl and De La Torre 2012). Interestingly, there was a modest yet significant increase in hydroxylamine oxidation in the presence of TETA. AOA, on the other hand, are believed to have a copper-dependent respiration pathway (Walker et al. 2010), aligning with the reduced hydroxylamine oxidation activity observed in the experiments in this study. Because key components of the archaeal electron transport chain require sufficient bioavailable Cu²⁺, chelation by TETA likely disrupts efficient electron flow from hydroxylamine oxidation. This impaired electron transfer would be expected to lower the overall catalytic turnover of hydroxylamine-processing enzymes, resulting in the diminished activity detected under copper-limited conditions (Vajrala et al. 2013). Hydroxylamine oxidation in AOB is mediated by hydroxylamine dehydrogenase (HAO), which can also oxidize hydrazine and is irreversibly inhibited by phenylhydrazine (Hooper and Nason 1965, Nishigaya et al. 2016). AOA lack a homolog of HAO (Walker et al. 2010), but they do oxidize hydrazine to N_2_, and hydroxylamine oxidation by AOA is inhibited by phenylhydrazine (Schatteman et al. 2022), suggesting some functional similarities between the ammonia/hydroxylamine oxidation pathways of AOA and AOB. Nonetheless, these findings indicate substantial differences in the trace-metal requirements of their hydroxylamine oxidation pathways.

To put our findings into an environmental context, the copper concentrations used in this study (ranging from nanomolar levels to 100 µM) span the gradient from copper-deficient to heavily contaminated soils. Total copper in pristine soils typically ranges from 2 to 40 mg/kg (approx. 30–600 µmol/kg), but the bioavailable fraction in soil pore water is often in the low micromolar or even nanomolar range due to complexation with organic matter (Flemming and Trevors 1989). Representatives of genus *Nitrosotalea* are predominate in acidic soils, whereas genus *Nitrosocosmicus* can be found in both neutral soils and acidic soils (Alves et al. 2018). The highest activity of AOB is typically found in neutral soils with high ammonia concentrations. Our results suggest that in contaminated soils where copper exceeds 5 µM, acidophilic AOA populations may face significant physiological stress, potentially shifting the community composition in favour of more tolerant species. For instance, *N. europaea* exhibited a high copper tolerance (up to 100 µM), which aligns with its frequent detection in environments with high metal loads, such as wastewater treatment plants or soils receiving long-term biosolids application (Sato et al. 1988, Giller et al. 1998, Park and Ely 2008). Given that low pH increases the bioavailability of free Cu^2+^, acidophilic AOA in acidic soils (pH < 5) are exposed to significantly higher effective copper stress than neutral-soil nitrifiers at equivalent total copper concentrations (Giller et al. 1998, Degryse et al. 2009). This suggests that *N. sinensis* is likely restricted to pristine or low-copper acidic niches, as even minor copper enrichment could reach toxic thresholds (Gubry-Rangin et al. 2011, Lehtovirta-Morley et al. 2011). Conversely, ‘*Ca*. N. franklandianus’, which demonstrated both high cellular copper content and resilience to copper deficiency, may possess superior copper acquisition and storage mechanisms. This would allow it to remain competitive in fertile arable soils where copper availability may fluctuate due to organic matter complexation (Lehtovirta-Morley et al. 2016, Alves et al. 2018).

The results presented in this study reveal some key differences between the physiology of archaeal and bacterial ammonia oxidizers. AOB rely on copper for the oxidation of ammonia, but their downstream oxidation pathway of ammonia is independent of copper. *Nitrosomonas europaea* (AOB) appears to have better adaptability to copper toxicity than AOA. However, despite being highly dependent on copper for oxidizing ammonia and hydroxylamine, '*Ca*. N. franklandianus’ was very resistant to copper deficiency, and it is likely to have some copper acquisition and storage mechanisms. In contrast, *N. sinensis* was much more sensitive to copper limitation. These effects could be further studied in the future by measuring cell densities. There remains a lack of knowledge about the possible competition of soil ammonia oxidizers for copper and whether copper availability or toxicity could act as niche-separating factors, as previously described for marine AOA and AOB (Shafiee et al. 2021), particularly given the complexity of soil, complexation of copper by organic matter, and uncertainty regarding metal bioavailability (Gwak et al. 2020). To further validate copper as a niche-defining factor, future field studies should move beyond measuring total soil copper. Quantifying the free Cu^2+^ fraction in soil pore water using techniques such as the Biotic Ligand Model (BLM) (Smolders et al. 2009) as well as experimentally amending the soil copper and organic matter concentrations will provide a more accurate reflection of the selective pressures shaping AOA and AOB communities. It remains unclear whether copper biofortification or foliar copper application influences soil nitrification processes and whether such practices could affect agricultural N₂O emissions. The differences in copper requirements among ammonia-oxidizing microbial groups challenge the use of nitrification inhibitors with a copper-chelating mechanism. The development of next-generation inhibitors could directly target hydroxylamine-oxidizing enzymes, as previously proposed (Schatteman et al. 2022).

## Supplementary Material

fiag026_Supplemental_File
