# Supplementary material for: Copper requirements and copper toxicity as niche-defining factors in the growth of terrestrial ammonia-oxidizing archaea and bacteria
Source: FEMS Microbiol Ecol. 2026 Mar 12;102(4):fiag026. doi: 10.1093/femsec/fiag026 (PMC13003922; doi:10.1093/femsec/fiag026)
Supplement: fiag026_Supplemental_File [file fiag026_supplemental_file.docx]

Supplementary materials

Table 1 Trace element content in growth media used for ammonia oxidisers. Medium A was used for ‘*Ca.* N. franklandianus’ and *N. europaea*. Medium B was used for *N. sinensis*. In order to remove copper, media were treated with Chelex resin applied either as 5 g/l or 50 g/l. Glassware used for the preparation of media was acid-washed. The presence or absence of the addition of Trace element mixture (TEM) is indicated in the third column. The limits of detection of the ICP-MS were 0.047 µg/l for iron, 0.036 µg/l for copper and 0.019 µg/l for zinc. Limits of quantification were 0.472 µg/l for iron, 0.357 µg/l for copper and 0.188 µg/l for zinc.

|  | Bottle | Chelex | TEM | Iron | Copper | Zinc |
| --- | --- | --- | --- | --- | --- | --- |
|  |  | [g l^-1^] |  | µg/l | µg/l | µg/l |
| Medium A | | | |  |  |  |
|  | Glass | 0 | no | 409.77 | 12.87 | 34.04 |
|  | Plastic | 5 | no | 5.35 | 2.10 | 2.71 |
|  | Plastic | 50 | no | 0.52 | 3.63 | 0.00 |
|  | Plastic | 5 | yes | 451.47 | 2.67 | 37.69 |
| Medium B | | |  |  |  |  |
|  | Glass | 0 | no | 153.25 | 4.30 | 41.91 |
|  | Plastic | 5 | no | 0.00 | 2.43 | 0.81 |
|  | Plastic | 50 | no | 4.10 | 3.31 | 0.88 |
|  | Plastic | 5 | yes | 399.23 | 1.99 | 31.21 |

Table 2 Predicted copper speciation in the medium A (10 nM added copper) of '*Ca.* N. franklandianus' and *N. europaea.* The speciation model was created using the visualMINTEQ software (Gustafsson, 2004).

| Species | concentration (M) | % of total Cu |
| --- | --- | --- |
| CuEDTA^2-^ | 10.00 × 10^-9^ | 99.97% |
| CuHEDTA^-^ | 2.39 × 10^-12^ | 0.02% |
| CuCO_3_ (aq) | 3.01 × 10^-13^ | 0.00% |
| CuHPO_4_ (aq) | 2.65 × 10^-13^ | 0.00% |
| Cu(OH)EDTA^3-^ | 1.30 × 10^-13^ | 0.00% |
| Cu^2+^ | **4.24 × 10^-14^** | **0.00%** |
| CuOH^+^ | 2.93 × 10^-14^ | 0.00% |
| Cu(NH_3_)^2+^ | 2.57 × 10^-14^ | 0.00% |
| CuHCO^3+^ | 5.53 × 10^-15^ | 0.00% |
| Cu(NH_3_)_2_^2+^ | 3.39 × 10^-15^ | 0.00% |
| CuCl^+^ | 3.20 × 10^-15^ | 0.00% |
| Cu(OH)_2_ (aq) | 1.65 × 10^-15^ | 0.00% |
| Cu(CO_3_)_2_^2-^ | 9.76 × 10^-16^ | 0.00% |
| Cu(NH_3_)_3_^2+^ | 1.24 × 10^-16^ | 0.00% |
| CuH_2_EDTA (aq) | 5.94 × 10^-17^ | 0.00% |
| CuCl_2_ (aq) | 5.14 × 10^-17^ | 0.00% |
| CuH_2_BO^3+^ | 1.43 × 10^-18^ | 0.00% |
| Cu(NH_3_)_4_^2+^ | 8.27 × 10^-19^ | 0.00% |
| Cu(OH)_3_^-^ | 1.87 × 10^-19^ | 0.00% |
| CuCl_3_^-^ | 1.96 × 10^-20^ | 0.00% |
| Cu_2_(OH)_2_^2+^ | 2.80 × 10^-23^ | 0.00% |
| Cu(H_2_BO_3_)_2_ (aq) | 4.13 × 10^-24^ | 0.00% |
| Cu(OH)_4_^2-^ | 3.06 × 10^-24^ | 0.00% |
| CuCl_4_^2-^ | 2.22 × 10^-24^ | 0.00% |
| Cu_2_OH^3+^ | 6.63 × 10^-27^ | 0.00% |
| Cu_3_(OH)_4_^2+^ | 1.56 × 10^-32^ | 0.00% |

Table 3 Predicted copper speciation in the medium B (10 nM added copper) of  *N. sinensis.* The speciation model was created using the visualMINTEQ software (Gustafsson, 2004).

| Species | Concentration (M) | % of total Cu |
| --- | --- | --- |
| CuEDTA^2-^ | 9.84 × 10^-9^ | 98.37% |
| CuHEDTA^-^ | 1.63 × 10^-10^ | 1.63% |
| CuH_2_EDTA (aq) | 2.05 × 10^-13^ | 0.00% |
| Cu^2+^ | 1.11 × 10^-13^ | 0.00% |
| CuHPO_4_ (aq) | 2.33 × 10^-15^ | 0.00% |
| CuOHEDTA_3_^-^ | 2.09 × 10^-15^ | 0.00% |
| CuHCO_3_^+^ | 8.65 × 10^-16^ | 0.00% |
| CuOH^+^ | 5.79 × 10^-16^ | 0.00% |
| CuCO_3_ (aq) | 5.60 × 10^-16^ | 0.00% |
| CuCl^+^ | 3.02 × 10^-16^ | 0.00% |
| CuNH_3_^+2^ | 6.11 × 10^-17^ | 0.00% |
| CuH_2_BO_3_^+^ | 3.94 × 10^-18^ | 0.00% |
| Cu(OH)_2_ (aq) | 2.32 × 10^-19^ | 0.00% |
| CuCl_2_ (aq) | 1.57 × 10^-19^ | 0.00% |
| Cu(NH_3_)_2_^+2^ | 7.44 × 10^-21^ | 0.00% |
| Cu(CO_3_)_2_^2-^ | 2.22 × 10^-21^ | 0.00% |
| Cu(H_2_BO_3_)_2_ (aq) | 1.37 × 10^-23^ | 0.00% |
| CuCl_3_^-^ | 2.71 × 10^-24^ | 0.00% |
| Cu(OH)_3_^-^ | 8.24 × 10^-25^ | 0.00% |
| Cu_2_(OH)_2_^2+^ | 2.48 × 10^-25^ | 0.00% |
| Cu(NH_3_)_3_^2+^ | 1.23 × 10^-26^ | 0.00% |
| Cu_2_OH^3+^ | 4.84 × 10^-28^ | 0.00% |
| CuCl_4_^2-^ | 2.23 × 10^-29^ | 0.00% |
| Cu(NH3)_4_^2+^ | 1.56 × 10^-30^ | 0.00% |
| Cu(OH)_4_^2-^ | 4.26 × 10^-32^ | 0.00% |
| Cu_3_(OH)_4_^2+^ | 1.65 × 10^-39^ | 0.00% |


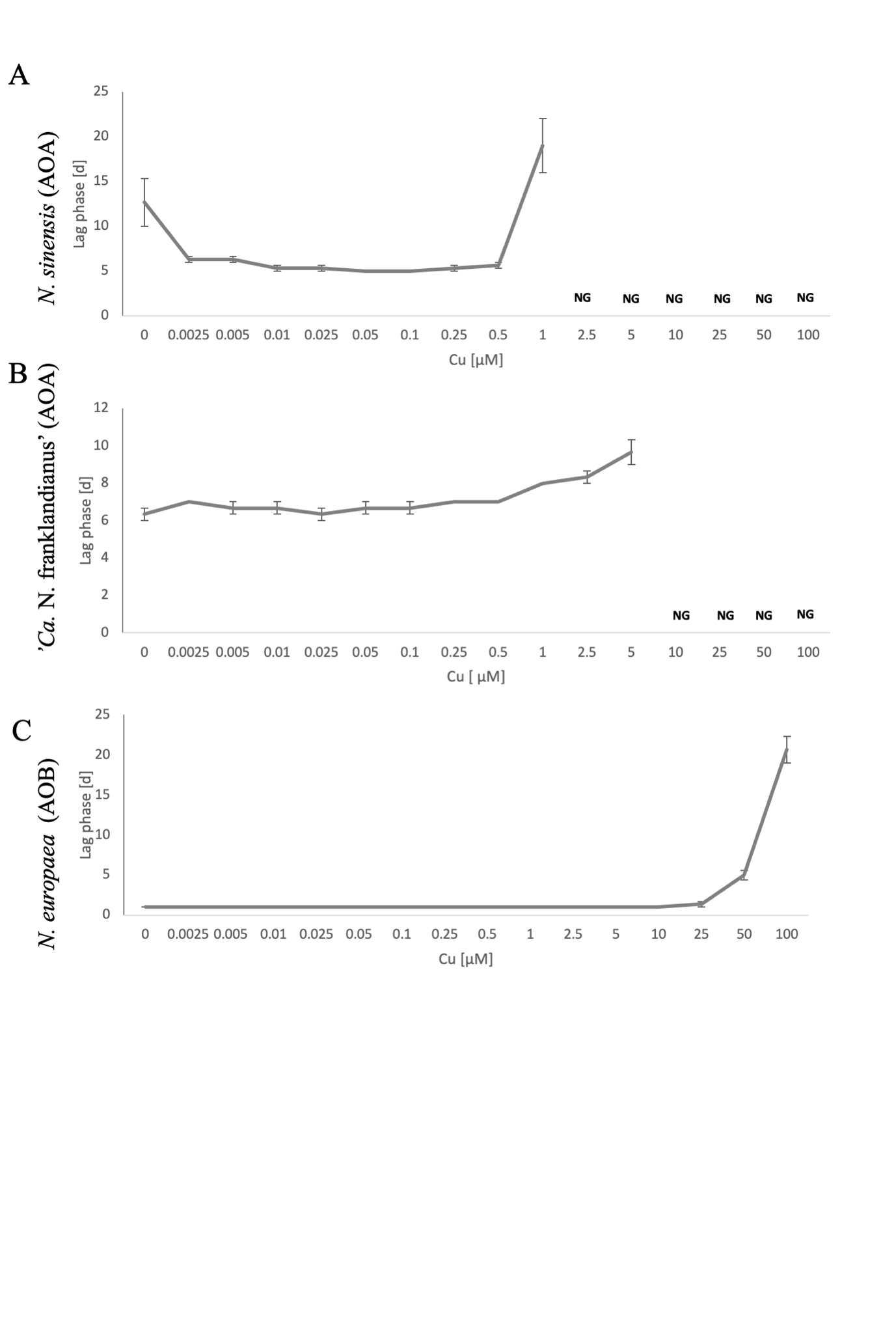


Figure 1 The effect of copper on the length of the lag phase during growth of *N. sinensis* (A), *Ca*. N. franklandianus (B) and *N. europaea.* Error bars represent standard error (n=3). NG signifies treatments which did not yield any signs of growth during the measuring period (30 days).
